# The Influence of the Gut Microbiota on Host Health: A Focus on the Gut–Lung Axis and Therapeutic Approaches

**DOI:** 10.3390/life14101279

**Published:** 2024-10-09

**Authors:** Amal S. Alswat

**Affiliations:** Department of Biotechnology, College of Science, Taif University, P.O. Box 11099, Taif 21944, Saudi Arabia; a.alswat@tu.edu.sa

**Keywords:** asthma, chronic obstructive pulmonary disease, fecal microbiota transplantation, probiotics, short-chain fatty acids

## Abstract

The human gut microbiota is a complex ecosystem harboring thousands of microbial strains that play a crucial role in maintaining the overall well-being of its host. The composition of the gut microbiota varies with age from infancy to adulthood and is influenced by dietary habits, environment, and genetic disposition. Recent advances in culture-independent techniques and nucleic acid sequencing have improved our understanding of the diversity of the gut microbiota. The microbial species present in the gut release short-chain fatty acids (SCFAs), which have anti-inflammatory properties. The gut microbiota also plays a substantial role in modulating the host′s immune system, promoting immune tolerance, and maintaining homeostasis. The impact of the gut microbiota on the health of the host is quite evident, as gut dysbiosis has been linked to various diseases, including metabolic disorders, autoimmune diseases, allergies, and inflammatory bowel diseases. The gut microbiota has bidirectional communication with the respiratory system, creating the gut–lung axis, which has been associated with different respiratory diseases. Therapeutic approaches targeting the gut microbiota, such as probiotics, prebiotics, dietary interventions, and fecal microbiota transplantation (FMT), aim to restore microbial balance and promote the growth of beneficial strains in the gut. Nonetheless, gaining knowledge of the complex interactions between the gut microbiota and the host is necessary to develop personalized medicine approaches and microbiota-based therapies for various conditions. This review summarizes studies related to the gut–lung axis with particular emphasis on the role of the microbiota. Future research directions are also discussed.

## 1. Introduction

Respiratory diseases include various disorders affecting the upper and lower respiratory tract. These diseases have high mortality rates [1] and can be caused by physiological or immunological imbalances or by microorganisms. Chronic respiratory ailments include asthma and chronic obstructive pulmonary disease (COPD), whereas pneumonia and tuberculosis are examples of microbiologically induced diseases [2]. Respiratory diseases impose a huge burden on global healthcare systems; estimates suggest yearly deaths of 3.9 million worldwide [3]. Respiratory ailments are influenced by a complex set of environmental, genetic, and lifestyle factors. For instance, different genetic predispositions and environmental factors trigger asthma [4], while COPD is primarily caused by exposure to tobacco smoke, certain fuels, dust, and chemicals [5]. The spread of infectious respiratory diseases has been hampered by globalization and mass travel [6].

Considering the high mortality and economic burden of respiratory diseases, different techniques have been developed for early detection, such as tomography (CT) and molecular diagnostic tests [7]. The latter, in particular, can indicate the involvement of microbial strains in respiratory outbreaks. Respiratory conditions are typically managed using bronchodilators, corticosteroids, and antibiotics [8], while recently developed therapies, such as probiotics, are expected to improve the treatment of severe asthma and other respiratory diseases [9].

Preventive measures are important in curbing respiratory ailments and infectious diseases. In addition to occurrence, prevention can reduce the severity of symptoms and aid in managing patients. Vaccination campaigns against influenza and pneumococcal infections have been found to be effective, particularly during the recent coronavirus outbreak [10]. Public awareness campaigns against smoking and reducing air pollution can play pivotal roles in reducing cases of chronic respiratory diseases [11].

In addition to the above-mentioned strategies, the modulation of the gut microbiota has also been reported to treat respiratory ailments [12]. The gut and respiratory system share an epithelial structure, as both evolved from a common ancestral line from the foregut and exhibit secretory immunoglobulin A (IgA) and goblet cells [13,14]. Indeed, they have been described as “biao-li” or related organisms in the Chinese literature [15]. This common ancestral origin has also been described considering the maturation of similar submucosal lymphoid tissues that play a pivotal role in acquired and innate immunity [13]. The involvement of the gut microbiota in the development of lung immunity, particularly in early life, has been established [16]. This association between the two organs has also been demonstrated by the fact that an aberration of gut microbiota in early life can lead to long-term respiratory problems [16]. Mesenteric lymph first enters the lungs and exposes the lung cells to lymph content prior to dilution with blood. The other lymph, intestinal lymph, finds its way into the systemic circulation through the thoracic duct, which transfers its contents to the subclavian vein and then flows to the right side of the heart before being pumped into the pulmonary circulation. This flow of lymph results in the pulmonary vascular bed being the initial point of contact with mesenteric lymph [16,17]. In addition to this lymph flow, the immune system also faces challenges due to injury to the intestinal mucosa that can generate harmful substances such as endotoxins, microbial metabolites, and hormones or inflammatory mediators. These molecules exert their action by decreasing the integrity of the gut barrier. After crossing the gut barrier, these substances enter the bloodstream through mesenteric lymph ducts and activate endothelial [18] and immune cells, ultimately resulting in acute lung injury (ALI) [18,19].

**Figure 1:**
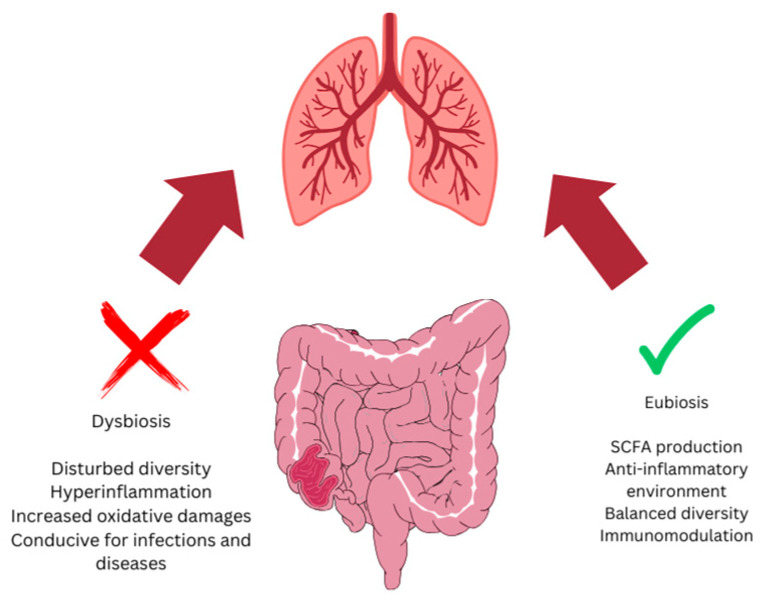
A balanced or healthy gut microbiota (eubiosis) harbors microbial species that release various metabolites, including short-chain fatty acids (SCFAs) with anti-inflammatory properties, and triggers immune cells that also maintain lung homeostasis. A disturbance in gut microbial species (dysbiosis) leads to various lung-related diseases.

Despite their similar origins and crosstalk through the immune system, the gut and lungs vary greatly in their microbial communities [13]. The two systems interact in various ways; gut microbes and their metabolites can interact with the respiratory system (Figure 1). Infants, at the time of birth, do not carry any significant microbial species; however, they acquire them from their mother and develop a particular repertoire during the first five years of their life [20]. However, this gut microbiota repertoire depends on various factors, including the delivery method, diet pattern, and other environmental factors [21]. Primarily, Firmicutes such as *Lactobacillus* and *Bacillus*, Bacteroidetes, Proteobacteria such as *E. coli*, and Actinobacteria dominate the gut microbiota. A change in the gut microbiota community is termed gut dysbiosis and has been linked to different physiological problems. For instance, earlier work indicated an association between the gut microbiota and lung diseases, as dysbiosis (Figure 1) has been associated with some respiratory diseases [22]. The fact that babies delivered through Cesarean surgery are prone to developing asthma [21,23] has affirmed a relationship between the gut microbiota and respiratory diseases, highlighting the gut–lung axis. Likewise, the use of antibiotics in infants can disrupt the gut microbiota and can reduce its diversity [24]. Epidemiological studies have also confirmed the association of early-life antibiotic use with an increased risk of asthma and other allergic diseases [20,24]. In addition to the direct and indirect roles of gut microbiota in lung health, gut microbial species can also find their way to the lung and shape the lung microbiota [25].

This review article aims to explore recent advances in the understanding, diagnosis, treatment, and prevention of respiratory diseases, particularly by highlighting the lung–intestine nexus and the role of the gut microbiota.

## 2. Human Gut Microbiota

The human gut microbiota is a community of billions of microbial strains, and it evolves after birth and changes according to the environment, lifestyle, and prevailing disease conditions [26]. It has been associated with a number of physiological phenomena and has been found to play a role in maintaining overall well-being. It is one of the most complex ecosystems on the planet, where several archaeal, prokaryotic, and eukaryotic species evolve together, exhibiting a variety of associations [22,26]. Recently, the availability of culture-independent techniques and advances in nucleic acid sequencing have improved our understanding of gut microbiota [13,26], and its role in physiological, psychological, and immunological processes has been established [13].

### 2.1. Composition and Diversity of the Gut Microbiota

The composition of the gut microbiota differs in the gastrointestinal tract [27]. The colon is the most enriched ecosystem, with diversified nutritional, biochemical, and microbial strains [22]. Members of Firmicutes and Bacteroidetes are predominantly found in the colon, while less abundant groups include Actinobacteria, Proteobacteria, and Verrucomicrobia [28]. However, the actual composition of the microbiome depends on dietary habits, environmental factors, age, and, most importantly, the genetics of the host [29]. Nonetheless, maintaining the diversity of a balanced microbiota is crucial for well-being, while an imbalanced microbiota is associated with different diseases [30].

### 2.2. Role in Metabolism

The gut microbiota primarily contributes to the gut ecosystem by releasing short-chain fatty acids (SCFAs) such as acetate, propionate, and butyrate [25]. SCFAs are products of the fermentation of indigestible dietary fibers and serve as energy sources for colonocytes [31]. SCFAs exhibit anti-inflammatory properties [32]. In addition to the catabolism of fibers, microbial species in the gut also produce some essential vitamins such as B vitamins and vitamin K, which are required for fat digestion and absorption [32]. Studies also indicate a role of gut microorganisms in the regulation of glucose and lipid metabolism, thereby triggering the risk of metabolic disorders, including obesity and type 2 diabetes [33].

### 2.3. Immune System Modulation

In addition to its niche, the gut microbiota also influences other body functions. Gut microbial species play a significant role in the development and operation of the host′s immune defense system and stimulate the maturation of immune cells [20]. They also release various peptides with antimicrobial activities, enabling the microbiota to maintain homeostasis in the gut [21,25]. Microbial antigens and metabolites interact with pattern recognition receptors on immune cells and formulate the immune response [27]. Crosstalk between the digestive and respiratory systems occurs through various signaling molecules. In particular, interleukin (IL)-25, IL-13, prostaglandin E2 and CD8+ T cells, and Th17 cells are induced by the gut microbiota and play a major role in the development of the host immune system [34]. Moreover, the interaction between the gut microbiota and lung flora also plays a role in lung diseases [25]. Some of the gut species that colonize the ileum can stimulate Th17 cells, leading to the activation of B cells in the lungs. This is of particular importance in autoimmune lung diseases [25]. The transfer of gut microbial species to the lung activates phagocytic cells, where antigen presenting cells (APCs) further modulate the lung immune system [35]. Additionally, the crossing of the blood barrier by gut microbiota can augment inflammatory reactions in the lungs through extra-intestinal T cell regulation [34].

This mechanism demonstrates how an appropriate and well-balanced microbiota promotes immune tolerance. However, dysbiosis can lead to immune dysregulation or even chronic inflammation, which contributes to autoimmune diseases, allergies, and inflammatory bowel diseases [36]. A change in the gut microbiota with an increased abundance of Clostridialis and Aerococcaceae has been associated with pulmonary hypertension [37]. In contrast, a higher number of beneficial bacteria, such as SCFA-producing strains, triggers regulatory T cells (T_regs_) and inhibits vascular inflammation [38]. Studies have suggested that SCFAs induce hematopoiesis in the bone marrow and affect the immune microenvironment of the lungs [27]. Digestive problems have also been reported to result in low levels of SCFAs and promote lung disease. SCFAs play their roles by reducing pH and facilitating mucin synthesis, thereby inhibiting the attachment of harmful bacteria [39]. The multifaceted roles of the gut microbiota have been recognized, as a decreased number of *Bifidobacterium* and Actinobacteria, along with an increased number of *Enterococcus* sp., has been associated with lung cancer [40]. Some gut species altered in dysbiosis can alter the neutrophil-to-leucocyte ratio, hence impacting the immune system [41]. Moreover, the gut microbiota has also been found to interact with the central nervous system, leading to the concept of the gut–brain axis [42]. Certain gut microbial species can release chemicals that act as neurotransmitters and influence brain function and behavior [43]. Alterations in the gut microbiota are reportedly associated with certain neuropsychiatric disorders, including depression, anxiety, and autism [44]. Therefore, a balanced gut microbiota is necessary to maintain overall mental well-being.

## 3. Relationship between Gut Microbiota and Respiratory Diseases

The nexus between two discrete anatomical and physiological systems, the gut and lungs, presents various mechanisms, including the movement of microbial metabolites, immune cell trafficking, and the systemic circulation of microbial components such as lipopolysaccharides (LPSs) and peptidoglycans [13,45]. These interactions indicate that the gut microbiota can influence lung health by modulating systemic immune responses and inflammation [22,28]. The regulation of the immune system under the influence of the gut microbiota impacts the balance between pro-inflammatory and anti-inflammatory responses [13,34]. Gut microbial species produce high levels of SCFAs that have anti-inflammatory properties and can enhance the function of regulatory T cells (T_regs_) [46]. T_regs_ migrate to the lungs and contribute to maintaining immune homeostasis, reducing the risk of inflammatory respiratory conditions such as asthma and chronic obstructive pulmonary disease (COPD) [45,46].

### 3.1. Influence on Respiratory Infections

The impact of the gut microbiota is not limited to respiratory diseases; rather, it has a role in elevating the susceptibility to and severity of microbial infections of the upper and lower respiratory tract [22,25]. Alterations in gut microbiota or dysbiosis can lead to the increased permeability of the gut barrier, thus increasing the chances of systemic inflammation and infections [29]. In contrast, a healthy and balanced gut microbiota limits the access of pro-inflammatory molecules and bacterial and viral pathogens to the respiratory tract. In addition, the metabolites produced by healthy gut microbiota have antiviral responses that protect the lung epithelial cells and offer protection against respiratory viruses such as influenza [47].

Viral infections have versatile effects on the gut microbiota, and, in turn, the gut microbiota plays different roles in viral pathogenesis. For instance, a change in the metabolites of the gut microbiota has been noticed upon infection by respiratory syncytial virus (RSV), in which the anti-inflammatory activity of sphingolipids, polyunsaturated fatty acids, and SCFAs increased [48]. The authors suggested that this shift in metabolites is a protective measure against viral infections. A similar shift in metabolites was observed for influenza A virus, indicating a common mechanism [49,50].

The association between gut microbial species and immune modulation through lipopolysaccharides (LPSs), lipoteichoic acid, and peptidoglycan by initiating the TLR pathway and activating specific immune cells has been reported previously [51]. Other researchers have reported the activation of intestinal Toll-like-receptors (TLR) by the gut microbiota as a potential mechanism contributing to influenza infection [52]. This observation affirmed an earlier finding, where LPS inoculation (mimicking gut modulation) rescued the immune system in an influenza mouse model [47]. The depletion of certain gut microbial genera has been associated with an increase in pro-inflammatory cytokines, such as tumor necrosis factor (TNF)-α, C-reactive protein (CRP), IL-6, and IL-10; therefore, this mechanism has been proposed as a common feature in many viral infections, including SARS-CoV-2 [53,54].

In the case of SARS-CoV-2, the role of the gut–lung axis was evidenced when the virus was detected in fecal samples and attributed to the expression of ACE2 receptors by enterocytes [55]. The ability of SARS-CoV-2 to infect gut bacteria under lab conditions has also been reported [56]. The progression and severity of pneumonia and acute respiratory distress syndrome in COVID-19 was also associated with the gut microbiota [57,58]. An association between the severity of COVID-19 symptoms and the gut microbiota was tentatively proposed by Tong et al. [59], as a decrease in SCFA-producing bacteria was noted in severe cases [60]. In addition to the anti-inflammatory role of SCFAs, the downregulation of ACE2 receptors by *Bacteroides* was also attributed to this link. A decrease in the butyrate-producing members of the families Ruminococcaceae and Lachnospiraceae has also been reported in severe COVID-19 infections [61]. A decline in the populations of *Faecalibacterium prausnitzii*, *Eubacterium rectale*, and *Bifidobacterium* species has also been reported in COVID-19 infection [61,62]. This led to the proposal of a diagnostic, prognostic, or even therapeutic role for gut microbial species in COVID-19 infection.

### 3.2. Relationship between Gut Microbiota and Lung Cancer

Lung cancer is highly prevalent and has a very high mortality rate [63]. Although some prognostic markers related to lung cancer have been identified, growing evidence suggests that the release of cytotoxic substances under the influence of gut dysbiosis is associated with lung cancers [25].

Laroumagne et al. [64] investigated bronchoscopy samples from 210 lung cancer patients and found a higher number of bacteria of gut origin, including high levels of Gram-negative bacilli such as *Escherichia coli* and *Enterobacter* [64], indicating that the gut microbiota crossed the barrier. In a later study, a high level of *Enterococcus* was found in the gut of patients with lung cancer [65]. The presence of high levels of *Actinobsacteria* spp. and *Bifidobacterium* spp. in the feces of lung cancer patients indicates a gut–lung axis [66]. The authors highlighted the use of *Enterococcus* and *Bifidobacterium* as potential biomarkers for lung cancer [65]. While working with non-small cell lung cancer (NSCLC) patients, Gui et al. [63] noticed a shift in the gut butyrate-producing bacteria, including *Clostridium leptum*, *Faecalibacterium prausnitzii*, *Ruminococcus*, and *Clostridial* cluster I. This shift in gut microbial species in lung cancer has been proposed as an area for further study in order to use these species as a cancer indicator, in conjunction with other blood-based or genetic biomarkers [67]. Shotgun genome sequencing of gut microbiota from lung carcinoma patients indicated reduced alpha diversity compared to the healthy study population [68]; the species of *Flavonifractor*, *Eggerthella*, and *Clostridium* were found in greater numbers in cancer patients.

The release of various metabolites by the gut community has been associated with lung carcinogenesis. Secondary bile salts produced by the gut bacteria reportedly have DNA-damaging abilities that can initiate cancer [69]. Among such metabolites, acetaldehyde [70] and deoxycholic acid [71] have been linked to the pathogenesis of esophageal and liver cancer. Moreover, a change in the microbial species in the gut may also lead to the release of free radicals, causing DNA damage and even initiating carcinogenesis [72].

In addition to the association between gut microbiota and lung cancer, studies have also suggested that certain gut microbiota influence the response to chemotherapy in lung cancer. Patients administered anti-programmed cell death protein (anti-PD-1) therapy respond positively if their gut microbiota is enriched with *Akkermansia muciniphila* species [73]. The same species was also positively correlated with the treatment response to immune checkpoint inhibitors (ICIs) used for lung cancer. Song et al. [74] transplanted fecal microbiota containing species of Proteobacteria, Firmicutes, Bacteroidetes, and Actinobacteria to improve the response to PD1. Likewise, another study used *Clostridium butyricum* to improve the response to ICI inhibitors and to prolong the survival of patients with NSCLC [75]. Similarly, oral feeding of *Lactobacillus acidophilus* in lung cancer mouse models treated with cisplatin demonstrated improved survival rates [76].

### 3.3. Relationship between Gut Microbiota and Chronic Obstructive Pulmonary Disease (COPD)

COPD is a progressive respiratory ailment characterized by congestion in the respiratory tract, mainly due to inflammation caused by long-term exposure to harmful particles or gases, particularly cigarette smoke [77]. Among the respiratory diseases, COPD is a leading cause of morbidity and mortality worldwide [1,3]. Since the gut microbiota plays a role in modulating immune responses and systemic inflammation, it also plays an important role in the progression of COPD.

COPD is primarily characterized by systemic inflammation, which contributes to its progression and comorbidities [77]. Gut microbial species release various metabolites and interact with human tissues, which play a significant role in mitigating systemic inflammation [46]. Gut dysbiosis alters the permeability of the gut barrier and allows the leakage of several microbial metabolites into the bloodstream [78]. Importantly, this results in the release of bacterial lipopolysaccharides (LPSs) that have antigenic properties [79]. The release of metabolites along with LPSs triggers systemic inflammation and aggravates COPD [46,79]. Studies have shown a significantly altered gut microbiota in patients with COPD. The abundance of beneficial bacteria such as *Bifidobacterium* and *Lactobacillus* is reduced in COPD patients, while pathogenic bacteria such as Enterobacteriaceae are more prevalent [80]. The change in microbial diversity plays a crucial role in elevating the levels of some systemic inflammatory markers, such as C-reactive protein (CRP) and interleukin-6 (IL-6); these markers have been associated with the severity of COPD [81].

In COPD, the gut–lung axis plays a significant role in modulating lung inflammation and immune responses. It has also been reported that dysbiosis can lead to reduced SCFA production [45,46,82], which impacts T_regs_ and anti-inflammatory markers [83].

The indiscriminate use of antibiotics also contributes to dysbiosis; however, in COPD patients, antibiotics are administered to manage primary and secondary bacterial infections that further aggravate dysbiosis and cause more severe COPD [77]. It is also imperative to note that the exacerbation phase in COPD patients necessitates the use of antibiotics [84].

Lifestyle factors, such as diets low in fiber and high in processed foods, hinder the ability of gut microbes to digest fiber, thus reducing SCFA production [46,78,85]. Consequently, the risk of systemic inflammation is increased, which impacts lung health. Smoking, a major risk factor for COPD, has been shown to alter the gut microbiota composition, reducing beneficial bacteria and increasing pathogenic bacteria [86]. Therefore, promoting a healthy and fiber-rich diet and discouraging smoking are potential strategies to modulate the gut microbiota and improve COPD outcomes.

### 3.4. Relationship between Gut Microbiota and Asthma

Asthma is a chronic and persistent respiratory condition manifested by the inflammation of the respiratory tract with elevated responsiveness that leads to recurrent obstruction of the airflow [87]. This repeated congestion can result in restlessness and hospitalization. Certain studies have shown that gut dysbiosis, particularly in early life, makes a person more prone to asthma [87], providing further evidence for the gut–lung axis. Hence, the establishment of the microbiota in early life is important for the development of a healthy immune system [78]. Studies also indicate that infants with less diversified gut bacteria are at an increased risk of developing asthma and other allergic diseases [20]. This phenomenon was further confirmed by studies in which infants with limited diversity in their gut microbiota appeared to be more susceptible to asthma and other allergic disorders [87].

Dysbiosis, specifically in terms of a decreased abundance of certain bacterial genera such as *Bifidobacterium* and *Lactobacillus*, has been associated with a higher risk of asthma [87]. Mechanistically, through the release of SCFAs, dysbiosis in asthma involves the differentiation of T_regs_, thus altering the gut barrier [88]. In another study, 92 children suffering from asthma and 88 healthy children were investigated. The data showed an abundance of *Akkermansia muciniphila* and *Faecalibacterium prausnitzii* in the healthy population [89]. These bacteria are speculated to suppress inflammation by modulating the secretion of interleukins. In contrast, the asthma population had higher levels of inflammatory factors, including C-reactive protein (CRP) and tumor necrosis factor alpha (TNF-α).

### 3.5. Relationship between Gut Microbiota and Cystic Fibrosis

Cystic fibrosis (CF) is a genetic disorder in which patients suffer from the production of thick mucus that obstructs the respiratory tract [90]. Consequently, patients with CF become susceptible to chronic respiratory infections and inflammation. Studies have shown that patients with CF have significantly altered gut microbiota compared to healthy individuals [91]. Although it may be a phenomenon of cause or effect, this dysbiosis is believed to have a significant role in both gastrointestinal and respiratory complications in CF patients [90]. CF patients frequently encounter the growth of pathogenic bacteria such as *Pseudomonas aeruginosa* in their gut as well as in their lungs [90,92]. A relationship between the gut and lungs, or gut–lung axis, was found, as the presence of *P. aeruginosa* in the gut elevates systemic inflammation and heightens the immune response, exacerbating respiratory symptoms [91]. This understanding of gut–lung interactions leads to the proposal of therapeutics for CF through dietary interventions and probiotics that not only modulate the gut microbiota but also positively impact lung health in CF patients by reducing inflammation and improving immune function [93].

### 3.6. Relationship between Gut Microbiota and Allergic Rhinitis

Allergic rhinitis, also known as hay fever, is an inflammatory condition of the nasal mucosa caused by allergens such as pollen, dust mites, and pet dander [94]. A relationship between the gut microbiota and the severity of allergic rhinitis has been described [95]. The imbalanced immune response promoted by dysbiosis has been linked to an increased risk of allergic diseases, including allergic rhinitis [94]. The modulation of the gut microbiota through probiotics has been shown to provide relief in the symptoms of allergic rhinitis by maintaining the immune system, particularly through the production of anti-inflammatory cytokines [95]. However, continued research is required to provide a customized composition of probiotics for particular respiratory ailments and infections.

## 4. Strategies for Gut Microbiota Modulation

The complex relationship between the gut microbiota and respiratory health has encouraged researchers to develop therapeutics with the potential to restore the microbiota and to ease the symptoms of respiratory ailments [96]. In this context, different strategies have been proposed and tested, including dietary modifications, probiotics, prebiotics, synbiotics, postbiotics, and fecal microbiota transplantation (FMT) [97]. The following sub-sections discuss these strategies and their implications for respiratory health.

### 4.1. Dietary Modifications

From birth, diet plays a pivotal role in the development and establishment of the gut microbiota. Diets rich in carbohydrates or proteins can promote the growth of fermentative or proteolytic species [26]. The evolution of modern society has impacted dietary habits; hence, the microbiota of urban individuals has been found to differ greatly from those of tribal people [98]. A fiber-rich diet supports the growth of good bacteria, as it alters the ratio of Firmicutes to Bacteroidetes and enhances the production of SCFAs [99]. SCFAs help to maintain gut barrier integrity, regulate immune responses, and reduce systemic inflammation [31]. Mice fed a high-fiber diet had an elevated level of SCFAs that enhanced immunity against influenza by inducing the differentiation of macrophages and dendritic cell progenitors (MDPs) to Ly6C monocytes, which patrol the airways and reduce pathogenesis [29]. In a trial conducted in Australia, an improvement in symptoms was observed after introducing inulin into the diet of individuals suffering from asthma [100]. The researchers noticed decreased levels of cell counts in sputum and other inflammatory markers, including IL-8 in blood. Moreover, upregulation of GPR41 and GPR43 was also observed and linked with improved lung function. Importantly, butyrate (an SCFA) can signal IL-10 production through the GPR109A pathway and promote steady-state immune homeostasis [101]. Likewise, propionate, another SCFA, works in a GPR41-dependent manner to trigger MDP cells and enhance phagocytic ability in the lungs [29].

In contrast, a fast-food-based urban diet, which is high in fat and sugar, can lead to gut dysbiosis and facilitate the establishment of pathogenic bacteria [102]. Indeed, individuals who switch from a high-fiber diet to an animal protein diet exbibit changes in their gut microbiota within 24 h [34]. A high-calorie diet disturbs the respiratory system, triggers LPS-induced pneumonia, and disturbs the Th17/T_reg_ balance [103]. Another study found that a lack of dietary fiber can enhance airway inflammation [104]. Therefore, promoting a balanced diet rich in fiber and nutrients is a fundamental strategy to modulate the gut microbiota.

### 4.2. Probiotics and Related Preparations

**Figure 2:**
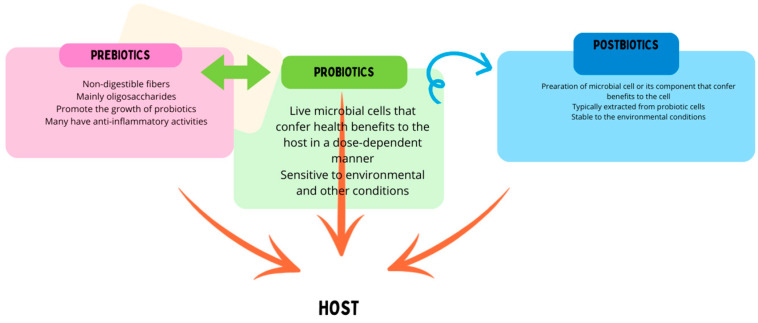
Probiotics, prebiotics, and postbiotics modulate the host immune system through various mechanisms. Prebiotics are used by probiotic strains for growth. Postbiotics are produced by (dead or inactive) probiotic strains with prebiotic molecules.

The microbiota associated with the human body has been recognized as an essential part of normal physiological function. Changes in microbial communities can lead to the development of many diseases, including GIT and lung-related diseases. Therefore, modulation of the microbiota through interventions such as the administration of probiotics has been described as “bio-therapy” (Figure 2) and has shown potential as a sole or adjuvant therapeutic agent [105].

#### 4.2.1. Probiotics

Probiotics have been available commercially for a long time [106] and are believed to provide health benefits to the host. Commonly used probiotic strains include *Lactobacillus*, *Bifidobacterium*, and *Saccharomyces* [107]. These strains are commonly used to treat gastrointestinal tract (GIT)-related problems. Probiotic strains facilitate the maintenance of a good gut environment by promoting a healthy gut microbiota. Probiotics also enhance gut barrier function and prevent the leakage of pro-inflammatory markers [108]. Thus, probiotics modulate immune responses and benefit respiratory health.

In addition to GIT-related problems, the therapeutic effects of probiotics on respiratory infections have also been investigated [12]. An earlier study demonstrated a reduced duration with less severity of the common cold in infants administered *L. gasseri* and *Bifidobacterium* [109]. In another study, the consumption of unpasteurized cow’s milk (which is rich in probiotic strains) was found to be associated with fewer episodes of asthma and allergic reactions in infants [110]. Considering the effect of probiotics on respiratory problems, researchers have already proposed the use of probiotics through the nasal route to augment the impact on lung-related diseases [111].

Probiotic strains, such as *Bifidobacterium* [111], *L. rhamnosus*, and *L. reuteri* [112], modulate the immune system by downregulating the expression of pro-inflammatory molecules such as IL-2, IL-6, and TNF-α and upregulating IL-10 and T_reg_ cells. This gut–lung crosstalk, aided by the “healthy microbiota”, establishes homeostasis at the lung mucosal level [113,114].

#### 4.2.2. Prebiotics

Prebiotics are non-digestible food ingredients that stimulate the growth and activity of beneficial gut bacteria [115]. Commonly used prebiotics include inulin, fructooligosaccharides (FOSs), and galactooligosaccharides (GOSs). By promoting the growth of beneficial bacteria, including probiotic strains, prebiotics have been shown to elevate SCFAs levels, improve gut barrier function, and support a balanced immune response [116]. For instance, supplementation with prebiotics in asthma patients has been associated with reduced inflammation and improved lung function [115,116]. Therefore, incorporating prebiotics into the diet can be a promising approach for promoting a healthy gut microbiota and respiratory health.

#### 4.2.3. Synbiotics

Synbiotics are a combination of probiotics and prebiotics designed to synergistically enhance the survival and colonization of beneficial bacteria in the gut [117]. Considering their composition, synbiotics can provide more substantial health benefits than probiotics or prebiotics alone. Studies have shown that synbiotics modulate the gut microbiota, increase SCFA production, and strengthen immune responses [117,118]. Similar to the use of probiotics, initial studies on the use of synbiotics for respiratory ailments and infections have provided promising results. A study demonstrated improved clinical outcomes in children with asthma, reducing the frequency and severity of asthma attacks after synbiotic supplementation [119]. The results of similar studies highlight the potential of synbiotics as a therapeutic strategy for respiratory diseases through gut microbiota modulation [119,120].

#### 4.2.4. Postbiotics

The International Scientific Association for Probiotics and Prebiotics (ISAPP) has defined postbiotics as preparations of killed or inactive microorganisms with or without components used for health benefits [121]. The components include cell wall molecules, proteins, SCFAs, polyamines, vitamins, and peptides including bacteriocins [122]. The exact composition depends on the source (probiotic strain). As a combination of various components, postbiotics modulate the immune system, inhibit harmful bacteria, improve the gut barrier, and establish immune homoeostasis [123,124].

#### 4.2.5. Fecal Microbiota Transplantation (FMT)

In addition to pro-, pre-, syn-, and postbiotics, FMT has also been proposed to restore a healthy gut microbiota, thereby reducing systemic inflammation and strengthening immune function. For instance, in a study using an animal model of chronic lung disease, FMT reduced inflammation in the lungs and improved lung function in the study population [125,126]. In another study, pneumonia was induced in gut microbiota-devoid mice; the transplant of fecal microbiota enhanced the function of alveolar macrophages, leading to decreased mortality in the study population [127]. Nonetheless, the use of FMT face ethical, religious, and safety issues; thus and hence, thorough experiments are needed to establish the efficacy and safety of FMT for respiratory disorders [125,128].

## 5. Clinical Studies on Gut Microbiota Modulation for Respiratory Health

This section reviews notable clinical studies that have investigated interventions by microbial species or by prebiotics, focusing on their efficacy and potential mechanisms.

Hao et al. [129] summarized clinical trial findings where probiotics reduced both the incidence and severity of respiratory infections, asthma symptoms, and COPD complications. Likewise, a randomized controlled trial showed that the administration of *Lactobacillus* and *Bifidobacterium* strains reduced the frequency of respiratory infections in children in daycare centers [130].

In another study, the effects of a combination of *Lactobacillus* and *Bifidobacterium* strains were evaluated in Cesarean-delivered children; researchers found a significant reduction in IgE-associated allergy and asthma symptoms, indicating the potential of probiotics in managing allergic conditions [131] (Table 1). In another randomized, double-blind, placebo-controlled trial, Chen et al. [132] found a reduction in the incidence of asthma exacerbations and an improvement in lung function in children administered *Lactobacillus rhamnosus* GG.

Schouten et al. [133] investigated the effects of prebiotics consisting of fructooligosaccharides in children with atopic dermatitis and asthma. Prebiotic supplementation improved gut microbiota composition, reduced allergic sensitization, and decreased asthma symptom severity (Table 2).

In another randomized, double-blind, and placebo-controlled study on prebiotic supplementation (galactooligosaccharides), Vulevic et al. [99] demonstrated that prebiotics significantly reduced the incidence of respiratory infections by enhancing immune function in elderly individuals. In their randomized controlled trial, de Boer et al. [134] assessed the effects of a synbiotic supplement (containing *Lactobacillus* and fructooligosaccharides) on children with asthma and found a substantial reduction in asthma attacks and improved overall asthma control. Li et al. [127] adopted a different approach by inducing chronic lung disease in mice through fine particulate matter and investigated the relieving effect of FMT. The authors reported a reduction in lung inflammation and improvement in lung function, suggesting potential therapeutic benefits for respiratory diseases.

In a phase IV trial registered with clinicaltrials.gov (NCT01301131), researchers at Mahidol University planned to study the impact of the *L. casei* Shirota strain containing fermented dairy product to prevent ventilator-associated pneumonia (VAP). However, the results of the study have not yet been published. In a phase II trial (registered as NCT05175833), researchers are investigating the effect of *Streptococcus salivarius* K12 with *L. brevis* on the prevention of pneumonia in long-COVID patients. In their phase I trial, Tian et al. [135] investigated the effect of *Clostridium butyricum* administration on lung cancer patients receiving chemotherapy. The use of the probiotic strain reduced the incidence of diarrhea and inflammatory response in patients. In another early phase I trial, Hua et al. [136] performed a multicenter study to investigate the positive impact of the oral administration of *L. rhamnosus* as an adjuvant therapy or influenza–pneumonia vaccine, along with inhaled amikacin, to treat patients suffering from acute exacerbation of COPD (AECOPD). The authors reported a significant reduction in the next episode of severe to moderate AECOPD in participants receiving either the vaccine or probiotics. In another early phase I trial (registration number NCT04857697), the authors described a study plan to investigate the effect of probiotics on breast or lung cancer patients undergoing surgery. In another clinical trial, Tranberg et al. [137] found that *Lactiplantibacillus plantarum* strains inhibited pathogens in vitro; however, they did not alter the oropharyngeal microbiota in patients.

**Table 1:** Clinical studies using probiotics to treat respiratory diseases.

Participants	Intervention	Outcome	Reference
Cesarean-delivered children	*Lactobacillus* and *Bifidobacterium* strains	Reduced IgE-associated allergy and asthma symptoms	[131]
Children with asthma	*Lactobacillus rhamnosus* GG	Reduced asthma exacerbations, improved lung function	[132]
Meta-analysis of RCTs	Various probiotic strains	Reduced incidence and duration of URTIs	[138]
Children in daycare centers	*Lactobacillus* and *Bifidobacterium* strains	Reduced frequency and severity of respiratory infections	[139]
Elderly individuals	*Lactobacillus casei* Shirota	Reduced incidence of respiratory infections	[140]
Patients with seasonal allergies	*Lactobacillus rhamnosus* GG	Reduced allergy symptoms	[141]
Healthy adults	*Lactobacillus* and *Bifidobacterium* strains	Reduced respiratory infection duration	[142]
Athletes	*Lactobacillus fermentum*	Reduced incidence of URTIs	[143]
Infants	*Lactobacillus reuteri*	Reduced respiratory tract infections	[144]
Children	*Lactobacillus reuteri* DSM 17938	Reduced respiratory infections	[145]

**Table 2:** Clinical studies using prebiotics and synbiotics for respiratory health.

Participants	Intervention	Outcome	Reference
Children with atopic dermatitis and asthma	Fructooligosaccharides	Improved gut microbiota, reduced asthma symptoms	[132]
Elderly individuals	Galactooligosaccharides	Reduced incidence of respiratory infections	[146]
Children with asthma	Synbiotic (*Lactobacillus* + FOS)	Reduced asthma attacks, improved control	[134]
Elderly individuals	Synbiotic (*Bifidobacterium* + inulin)	Enhanced immune response, reduced infections	[147]
Infants	Synbiotic (*Bifidobacterium* + GOS)	Reduced incidence of respiratory infections	[140]
Pregnant women	Synbiotic (*Lactobacillus* + GOS)	Reduced allergic disease in offspring	[148]
Healthy adults	Synbiotic (*Lactobacillus* + inulin)	Improved immune function, reduced infections	[149]
Children	Synbiotic (*Lactobacillus* + FOS)	Reduced respiratory infections	[150]
Infants	Synbiotic (*Lactobacillus reuteri* + GOS)	Reduced incidence of infections	[151]
Elderly individuals	Synbiotic (*Bifidobacterium* + inulin)	Enhanced immune response	[152]
Infants	Synbiotic (*Lactobacillus* + GOS)	Reduced respiratory symptoms	[24]

## 6. Future Directions, and Challenges in Investigating the Gut–Lung Axis

An expanding understanding of the gut–lung axis has revealed new paths to manage and treat respiratory diseases. However, various challenges remain in the development of novel therapeutics. A critical future direction in gut–lung axis research should focus on exploring the mechanisms underlying bidirectional communication between the gut and the lungs. This will allow researchers to explore the molecules responsible for the establishment of healthy gut microbiota.

Considering the role of the gut–lung axis in modulating the immune system, future research should decipher the role of biomarker signals with immune cells. The understanding of gut-derived regulatory T_regs_ is still in its infancy, and studies in this regard can provide new therapeutic targets [139]. Another neglected research area includes the role of gut-associated lymphoid tissue (GALT) in respiratory immunity, the knowledge of which is essential to uncover novel immune pathways linking gut health to lung function [153].

Understanding the role of microbial metabolites other than SCFAs [154] is also lacking; hence, a broader perspective on the effect of microbial metabolism on gut and lung health is yet to be captured. In this context, advanced metabolomics can assist in uncovering metabolites and their pathways, which can lead to the identification of new biomarkers and therapeutic agents [155].

It is evident that the personalization of gut microbiota-based therapies will provide an effective treatment option; however, research focusing on the above or a para-microbiota approach is required in this regard. The individuality of gut microbiota composition also suggests the need for a tailored approach rather than a one-size-fits-all strategy. A holistic research approach is needed to develop personalized medicine that considers an individual′s microbiota profile, genetic background, and lifestyle [156].

Well-studied probiotic strains have already been tested for respiratory diseases; however, new and less studied strains should be included in clinical trials to broaden the therapeutic landscape. There is little understanding of the optimal dose of pro- and prebiotics, particularly synbiotics [157]. The development of new synbiotic formulations will also provide new avenues for future research.

Next-generation sequencing (NGS) and advancements in this field have revolutionized the science of microbiology at the microbiota level. NGS-based techniques, along with proteomics and metabolomics tools, will enable researchers to understand the gut microbiota [158] and its interaction with the lungs and other organs. Advanced imaging techniques in conjunction with artificial intelligence (AI)-based detection tools can assist researchers in understanding the functions of the microbiota at the organ and tissue levels.

Innovations in imaging technologies, such as two-photon microscopy and positron emission tomography (PET), can provide insights into changes in tissues and cells under the influence of the gut microbiota. This will also deepen our understanding of the temporal dynamics of the gut–lung axis, offering unprecedented insights into the complex interplay between the gut microbiota and lung tissues [159].

AI and machine learning (ML) have made the processing of large datasets possible and have the potential to explore tissue- or organ-specific microbial species with an impact on the immune system. AI-based models can help to develop predictive biomarkers for respiratory diseases and tailor personalized treatments based on individual microbiota profiles [160].

Currently, microbiota-based studies are cross-sectional and, hence, provide limited data. Comprehensive longitudinal studies are vital to explore the gut–lung axis and to detect dynamic changes in the gut microbiota and their impact on respiratory health over time. Long-term studies observing cases from infancy to adulthood from multi-center trials involving diverse populations could provide concrete evidence of the microbial role in the gut–lung axis [161,162]. Standardization of methodologies and protocols, including sample collection, storage, and analysis, is essential to ensure the reproducibility and comparability of results across studies [163]. These studies also require collaborative efforts among researchers, funding agencies, and regulatory bodies to develop and implement these standards [164].

Research related to the use of probiotics and FMT also faces challenges due to ethical considerations. Ensuring informed consent, protecting participant privacy, and addressing the potential risks associated with interventions are critical ethical challenges that can be addressed through mass awareness [165].

## 7. Conclusions

The human gut microbiota is a complex ecosystem influencing metabolism, immune modulation, and susceptibility to acquiring allergic responses and microbial infections. The concept of the gut–lung axis highlights the involvement of gut microbiota in several respiratory problems, including asthma, chronic obstructive pulmonary disease (COPD), influenza, and pneumonia. Considering the prevalence and high mortality rates of respiratory diseases, the gut microbiota has been investigated for its possible role and therapeutic effects in respiratory ailments and infections. In this context, the presence of “good microbiota”, or eubiosis, has been associated with gut and lung well-being, whereas a disturbed microbiota, or dysbiosis, leads to several gut and lung disorders, including lung cancers. The therapeutic approaches involving probiotic strains with prebiotics (synbiotics) or postbiotics have been largely recognized. However, further studies are required to develop personalized probiotic strains for better and effective treatment.
